# Enhancing Viability of *Lactobacillus plantarum* BG24 Through Optimized Spray Drying: Insights Into Process Parameters, Carrier Agents, Comparative Analysis With Freeze Drying, and Storage Condition Influences

**DOI:** 10.1002/fsn3.4572

**Published:** 2024-11-07

**Authors:** Figen Kaymak Ertekin, Özgün Köprüalan Aydın, Özgül Altay

**Affiliations:** ^1^ Department of Food Engineering, Faculty of Engineering Ege University İzmir Turkey

**Keywords:** carrier agents, cell viability, freeze drying, *Lactobacillus plantarum* BG24, spray drying, storage

## Abstract

This study investigated the survival dynamics of *Lactobacillus plantarum* BG24, a probiotic strain, within reconstituted skim milk (RSM) and yeast extract (YE) matrices during the spray–drying (SD) process, encompassing of inlet/outlet air temperatures. Notably, optimum SD parameters were found to be an inlet air temperature of 150°C and outlet air temperature of 83°C, that achieving high viability (92.23%), and reducing both moisture content (MC) (3.57%) and water activity (*a*_w_) (0.266). The use of soy protein isolate (SPI), gum Arabic (GA), RSM, maltodextrin (MD), sucrose (SUC), and lactose in binary mixtures or alone was investigated in terms of the best survival rate of probiotic bacteria, and RSM alone and RSM + GA and SPI alone were found to be the best drying carriers giving higher viability during SD. SD at optimum process temperatures and freeze drying (FD) were compared in the survival rate of probiotic bacteria in the carrier of RSM with YE, and FD samples showed a higher survival rate (97.69%) than SD samples. It was determined that the storage temperature (4°C and 20°C) had an impact on the glass transition temperature, MC, *a*_w_, and cell viability. Increased storage temperature led to a greater decrease in cell viability, especially for SD probiotic powders. These findings furnish critical insights into the intricate interplay among process parameters, carrier agents, drying techniques, and storage conditions, thereby elucidating avenues for refining probiotic preservation strategies within the ambit of SD, and by extension, in the domains of food and pharmaceutical sciences.

## Graphical abstract

The optimum SD conditions were found to be at 150°C inlet and 83°C outlet temperatures, with higher viability. The viability of RSM, RSM + GA, and SPI powders was higher during spray drying. Cell viability increased when *L. plantarum* BG24 powder was stored at 4°C.

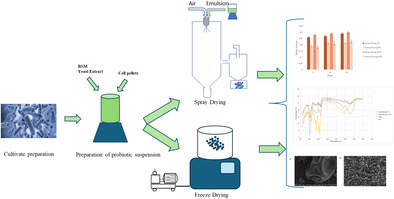

## 1 Introduction

Probiotics, live microorganisms beneficial for human health, have been extensively studied over the past century due to their ability to prevent and manage conditions like diabetes, stomach cancer, and inflammatory bowel disease (Razavi et al. 2021). They achieve this by strengthening the gut barrier, producing antimicrobial substances, and boosting immune defenses. People are increasingly interested in incorporating probiotics into their diets through food products or supplements, driven by a desire to strengthen their immune systems naturally. *Lactobacillus and* Bifidobacteria groups are commonly used probiotics, with other species like *Bacillus cereus* and nonpathogenic strains of *Escherichia coli* also showing promise (Solanki et al. 2013). Among these strains, *Escherichia coli* Nissle 1917 (EcN) is particularly noteworthy due to its advantageous probiotic properties and the ease with which it can be genetically manipulated (Zhao et al. 2022). For a century, probiotic *Escherichia coli* strain Nissle 1917 has been used to treat a variety of intestinal illnesses (Nougayrède et al. 2021; Pradhan and Weiss 2020). *Lactobacillus plantarum*, in particular, is a versatile species found in various ecological habitats, including dairy products, and is widely used as a starter culture in fermented foods and beverages due to its favorable taste and texture (Gökmen et al. 2024). In a study conducted by Meena et al. (2022), the isolation and probiotic properties of *Lactiplantibacillus plantarum* from traditionally fermented cereal‐based food products in India (such as Makka ki Raab, Olia, Jalebi batter, Raabadi, and Kadhi) were investigated. The study showed that potential probiotic strains could be used as starter cultures for the commercial preparation of indigenous cereal‐based products and other futuristic functional foods for the broader public benefit. In another study conducted by Meena et al. (2024), corn flour and skim milk powder (SMP) were used to develop a probiotic milk‐cereal‐based food powder. A milk‐cereal‐based probiotic drink, Raabadi, was developed using the probiotic *Lactobacillus helveticus* MTCC 5463; the drink was spray dried to produce probiotic powder, and process conditions were optimized. It was reported that the optimized drink powder, which was nutritionally rich in protein, also contained a good amount of phenolic, antioxidants, and minerals. Furthermore, this method has the potential to be adopted and scaled up for the commercial production of milk‐cereal‐based spray‐dried probiotic, Raabadi powder. In a study conducted by Gökmen et al. (2024), the probiotic properties and optimal growth conditions of *Lactiplantibacillus plantarum* BG24, isolated from fermented boza beverage, were investigated, and *L. plantarum* BG24, developed under the identified optimal growth conditions, was concluded to be beneficial for lactose–intolerant patients due to its β‐galactosidase activity. Furthermore, the data obtained from this study are expected to guide other researchers seeking new alternative probiotic lactic acid bacteria.

Probiotics need to be metabolically active in sufficient amounts during passage through the stomach and intestine to provide health benefits. Maintaining a minimum concentration of 10^6^–10^8^ colony‐forming units (CFU)/g in the product is essential for their positive effects (De Prisco and Mauriello 2016; Rezazadeh‐Bari et al. 2019). However, probiotics are sensitive to adverse conditions, making it challenging to preserve their viability during production, shelf life, and gastrointestinal transit. Various drying methods, such as freeze drying (FD) and spray drying (SD), are commonly used for microorganism preservation. FD prevents degradation during storage, but it is costly (Morgan et al. 2006). In contrast, SD is cost‐effective and allows for the production of large quantities of stable powders. However, high processing temperatures in SD can damage heat‐sensitive microorganisms, necessitating careful consideration of factors like strain type, drying temperature, and storage conditions to ensure effective preservation (Gardiner et al. 2002).

Drying of probiotic microorganisms is a critical step in enhancing the stability of these health‐friendly components and facilitating their integration. The selection of environments during this process influences crucial factors such as organism preservation, control of water components, drying speed and efficiency, preservation of nutritional values, and applicability (Corcoran et al. 2004). The primary cause of viability loss during the drying process is attributed to alterations in the physical state of membranous lipids, which lead to the disruption of membrane integrity and fluidity. Enhanced cell viability and minimal structural damage can be achieved by incorporating simple or complex substances such as sugars, skim milk, whey, amino acids, dietary fibers, and glycerol, some of which possess prebiotic properties (Araújo et al. 2020; Meena et al. 2023). These matrices function mainly to protect microbial cells by mitigating the detrimental effects of dehydration.

Therefore, the vital selection of an appropriate environment is essential for effective drying of probiotics. During this process, a variety of protective agents have been included to enhance the viability of bacteria. These include whey protein, trehalose, monosodium glutamate, gum Arabic (GA), glycerol, betaine, adonitol, SUC, glucose, inulin, lactose, and oligosaccharides. Researches indicated that reconstituted skim milk (RSM) powder serves as a suitable carrier medium for the SD of probiotic cultures (Ananta, Volkert, and Knorr 2005; Corcoran et al. 2004; Desmond, Stanton, et al. 2002; Meena et al. 2023; Morgan et al. 2006; Sunny‐Roberts and Knorr 2009). The high survivability of probiotic cells can be attributed to the presence of lactose and various fractions of milk proteins, such as caseins, α‐lactoglobulin, β‐lactoglobulin, bovine serum albumin, and lactoferrins, which help prevent and minimize structural cell damage, thereby protecting cellular function during dehydration (Mis Solval et al. 2020). Additionally, some researchers have reported interactions between lactose and the polar groups of phospholipids and proteins in the bacterial cell membrane, while milk proteins help reduce membrane leakage and maintain cellular integrity, thus minimizing cellular injury and inactivation during SD (García 2011; Meena et al. 2023; Santivarangkna, Higl, and Foerst 2008).

For instance, it has been found that *Lactobacilli* preserved with SD in RSM exhibit good preservation (Ananta, Volkert, and Knorr 2005). Certain polymers are expected to provide minimal protection when they do not interact with proteins or cell membranes. Desmond, Ross, et al. (2002) found that the use of 10% GA and 10% RSM rather than 20% RSM increased *L. paracasei* survivability during SD and storage for up to 4 weeks. In another study by Tang et al. (2020), the effectiveness of SD and FD methods was compared, and the effects of adding of various protectants, such as skim milk, SUC, maltodextrin (MD), and cornstarch, on the preservation of *Lactobacillus acidophilus* FTDC 3081 cells were investigated. After 1 month of storage, MD was reported to show a higher protective capability in both spray‐dried and freeze‐dried cells compared to other protectants. The use of RSM + MD yielded the best results compared to the use of MD alone. A complete loss of viability was observed for spray‐dried *L. acidophilus* FTDC 3081 cells at 40°C after 1 month of storage in the absence of any protectant. Some sugars, particularly disaccharides, are known to replace water molecules to protect membrane structures. These protectants have been observed to enhance cell recovery by preserving proteins and lipid membranes during storage. However, each protective agent has its disadvantages and is typically combined to form a special formulation. For instance, some studies have demonstrated that the *Lactobacillus casei* LC15 strain can achieve a survival rate of 82.2% during FD (Chen et al. 2008). Additionally, some studies have shown that a combination of trehalose, skim milk, and manganese sulfate has been shown to increase the survival rate of *L. plantarum* to 96.8% ± 1.3% (Li et al. 2012).

This study aims to enhance the probiotic viability during the drying and storage processes of the *L. plantarum* BG24 strain isolated from boza. Toward this end, the optimization of SD process conditions, investigation of various protective environments on the postdrying stability of dried probiotics, and determination of storage stability of microorganisms obtained through SD and freeze‐drying (FD) methods are intended. This research will be a significant step in preserving the health benefits and ensuring long‐term utilization of *L. plantarum* BG24, potential probiotic microorganism derived from boza.

## 2 Material and Methods

### 2.1 Microorganism and Cultivate Preparation

The study utilized the potential probiotic strain *L. plantarum* BG24, which was isolated from the naturally fermented cereal beverage, boza. This strain was produced on a laboratory scale at the Ege University, department of food engineering (Gökmen et al. 2024). The *L. plantarum* BG24 strain underwent incubation in a 100 mL MRS (De Man Rogosa Sharpe) Broth medium with a 10% inoculum at 37°C for 24 h. After the incubation period, the microorganism was separated from the medium through centrifugation at 4000 rpm for 10 min at 4°C. Subsequently, it underwent two washes with peptone water to obtain the bacteria in a wet pellet form.

### 2.2 Preparation of Probiotic Suspension for Spray‐Drying Process

The wet cell pellets were re‐suspended in RSM (20% w/v; Oxoid, Hampshire, UK) and yeast extract (YE; 0.5% w/v; Sigma Aldrich, USA) prepared in phosphate buffer (pH: 7.2). Phosphate buffer was prepared in the proportions specified by Broeckx et al. (2017). The RSM and YE prepared in phosphate buffer were sterilized at 115°C for 10 min (Zayed and Roos 2004), and stored at +4°C for further study. Before SD, the probiotic mixture was re‐suspended for 5 min using a magnetic stirrer and fed to the system. The number of viable cells in carrier solutions (CFU/mL) prior to SD and in powder (CFU/g DM) after SD was determined. All microbiological and physical analyses were completed on the day of drying.

### 2.3 Spray Drying

The probiotic suspension was dried in a lab‐scale spray drier (Buchi Mini Spray Drier B‐290, Buchi Labortechnik, Switzerland) with a constant aspiration rate (60%) of the drying air, an airflow rate of 473 L/h, an air pressure of 5 kg/cm^2^, and a 0.7 mm diameter nozzle. The suspension was fed into the spray dryer at 25°C via a peristaltic pump. The objective of maintaining these consistent SD conditions was to reduce the amount of *a*_w_ and moisture content (MC) while increasing the survival rate. As independent process variables, inlet temperature (145°C –180°C) and outlet temperature (60°C–85°C) were selected. At outlet of the spray dryer, a cyclone separator was used to collect the dry powder. The dried samples were hermetically sealed in aluminum‐laminated polyethylene (ALPE; 12 μm PET + 8 μm AL + 50 μm LDPE) 110 × 210 mm pouches and stored at 4°C until the analysis.

### 2.4 Experimental Design and Statistical Analysis

The independent process variables, specifically the inlet temperature (ranging from 145°C to 180°C) and outlet temperature (ranging from 60°C to 85°C), were chosen for investigation. The impact of these process parameters on the survival rate, MC, and *a*_w_ of probiotic powder was examined. This exploration was conducted through the implementation of the CCRD (Central Composite Rotatable Design) experimental design, involving 13 runs. The results, presented in Table 1 as averages, were derived from duplicate experiments. Statistical software (Design Expert‐version 12 by Stat‐Ease Inc.) was employed for the evaluation of the obtained results.

**TABLE 1:** Viable cell counts, survival rate, water activity, MC, drying yield, and densities of *Lactobacillus plantarum* BG24 after SD at different inlet and outlet air temperatures according to central composite rotatable experimental design.

Run	Inlet air temperature (A)/outlet air temperature (B) (°C)	Viable cell counts (log CFU/g DM) Before drying	Viable cell counts (log CFU/g DM) After drying	Survival rate (%)	MC (%, wb)	Water activity	Drying yield (%)	Bulk density (kg/m^3^)	Tapped density (kg/m^3^)
1	163/73	9.68 ± 0.14	9.14 ± 0.17	94.46	5.96 ± 0.39	0.380 ± 0.003	77.09	392.50 ± 22.63	468.06 ± 25.34
2	150/64	9.54 ± 0.02	9.34 ± 0.08	97.94	6.74 ± 0.23	0.400 ± 0.003	72.91	334.88 ± 20.49	414.76 ± 27.74
3	163/60	9.72 ± 0.05	9.69 ± 0.05	99.73	8.75 ± 0.27	0.475 ± 0.002	70.99	265.39 ± 16.05	377.93 ± 25.67
4	175/81	9.54 ± 0.31	8.96 ± 0.29	93.90	5.39 ± 0.55	0.360 ± 0.018	74.68	327.11 ± 14.44	405.06 ± 5.95
5	145/73	9.76 ± 0.01	9.27 ± 0.19	95.00	4.55 ± 0.28	0.298 ± 0.001	79.06	394.69 ± 20.51	427.33 ± 22.47
6	163/73	9.68 ± 0.26	9.11 ± 0.13	94.02	5.84 ± 0.23	0.385 ± 0.011	77.63	354.69 ± 23.36	430.53 ± 40.13
7	163/73	9.62 ± 0.08	9.16 ± 0.10	95.19	5.58 ± 0.20	0.382 ± 0.007	76.37	366.91 ± 22.29	429.98 ± 7.25
8	180/73	9.78 ± 0.03	9.35 ± 0.33	95.63	7.23 ± 0.26	0.443 ± 0.007	72.62	332.38 ± 27.81	402.47 ± 8.07
9	163/85	9.64 ± 0.29	8.92 ± 0.24	92.54	3.79 ± 0.12	0.29 ± 0.007	79.06	406.76 ± 17.67	455.18 ± 20.38
10	163/73	9.94 ± 0.03	9.27 ± 0.09	93.24	5.79 ± 0.16	0.391 ± 0.004	77.28	377.30 ± 8.01	446.42 ± 13.12
11	175/64	9.67 ± 0.15	9.60 ± 0.24	99.20	9.30 ± 0.31	0.510 ± 0.006	68.94	235.53 ± 6.53	320.03 ± 14.76
12	150/81	9.78 ± 0.13	9.00 ± 0.38	92.01	3.87 ± 0.33	0.290 ± 0.004	80.52	416.77 ± 9.32	507.44 ± 27.56
13	163/73	9.80 ± 0.03	9.27 ± 0.02	94.56	5.69 ± 0.17	0.399 ± 0.002	77.19	330.50 ± 13.90	388.05 ± 5.67

*Note:* Values are the mean ± SD of two independent assays.

Upon constructing a mathematical model, each response model is tested through multiple linear regression analysis. Analysis of variance (ANOVA) is then employed to discern the significant variables within each model, aiding in the identification of crucial factors influencing the outcomes. This study's goals were to minimize *a*_w_ (within the range of 0.1–0.25), minimize MC, and maximize survival rate by the use of both the desirability function (Derringer and Suich 1980) and the graphical techniques (superimposing; Koç and Kaymak‐Ertekin 2009).

Equation (1), which is predicated on the assumption of a quadratic model, illustrates how optimization is utilized to forecast responses in this investigation:

(1) $$R=\beta_{0}+\sum_{i=1}^{k}\beta_{i}x_{i}+\sum_{i=1}^{k}\beta_{\mathrm{ii}}x_{i}^{2}+\sum_{i=1}^{k-1}\sum_{j=i+1}^{k}\beta_{\mathrm{ij}}x_{\mathrm{ji}}\left(k=1,2,3\right)$$

where *R* is the predicted response, *β*_o_ is the constant coefficient, *β*_*i*_ is the linear coefficient, *β*_*ii*_ is the quadratic coefficient, *β*_*ij*_ is the interaction coefficient, and *k* is the number of independent variables. The model's accuracy of fit was assessed using ANOVA and *R* ^2^ values.

The model predicted an optimum point, or the optimal processing conditions, and five trials were conducted to verify the optimum point experimentally. To validate the optimum point for all responses, the single sample *t*‐test and single sample ANOVA (Duncan, post hoc) were employed (*p* < 0.05). These statistical tests assessed whether a statistically significant difference existed between the mean results predicted by the model and those obtained from the experiments. The SPSS 15.0 Windows packaged program (SPSS Statistical Software, Inc., Chicago, IL, USA) was utilized for conducting these statistical analyses.

### 2.5 Different Carrier Materials for Spray Drying

Under the optimal conditions of the SD process, various carrier materials were tested to achieve the highest survival rate and the lowest MC and a_w_ for dried probiotic bacteria. *L. plantarum* was suspended in distilled water dispersions with 20% (w/v) concentration of each of the following carrier materials: RSM + GA (Tito, İzmir, Türkiye); RSM + MD (DE20) (Tito, İzmir, Türkiye); RSM + SUC (Tito, İzmir, Türkiye); soy protein isolate (SPI) (Smart Kimya San., Türkiye); SPI + MD (DE20); SPI + Lactose (LAC) (Smart Kimya San., Türkiye); SPI + SUC. When combined, the protein‐to‐carbohydrate ratio was maintained at 1:1.

The dried samples consisting of *L. plantarum* BG24 culture with the highest survival rate were enclosed in hermetically sealed ALPE pouches. Subsequently, it was stored at 4°C for a maximum duration of 2 days before undergoing analysis.

### 2.6 Freeze Drying

After selecting the most suitable carrier agents that provide the highest survival rate for the probiotic suspension during the SD, the suspension, prepared with the same formulation, was frozen at −18°C and freeze dried at −55°C under a vacuum pressure of 0.15 mbar for 18 h (Telstar, Lyoquest Lyofilizator, Spain). Before analysis, the dried powder was kept in ALPE packets that were hermetically sealed and kept at 4°C for a maximum of 2 days.

### 2.7 Storage Stability

Samples of the freeze‐dried and SD probiotic powders were packaged under vacuum and kept at +4°C and 20°C (room temperature) for 30 days to evaluate the probiotic powders’ storage stability. Every 15 days, samples were taken out and counted for the determination of the survival rate, *a*_w_, and MC.

### 2.8 Analysis

#### 2.8.1 Moisture Content and Water Activity

According to AOAC (2005), the MC of powders that were FD and SD was ascertained using an oven set at 105°C. Using an *a*_w_ meter (Testo AG‐400, Germany) with an accuracy of ±0.001, the *a*_w_ of the powders was measured in duplicate at room temperature (Khem, Small, and May 2016).

#### 2.8.2 Drying Yield

After each SD trial, the drying yield (%) was determined by dividing the initial quantity of solids in the liquid feed by the percentage of powder weight collected from the receiving jar attached to the bottom of the cyclone (Shi, Fang, and Bhandari 2013).

#### 2.8.3 Cell Viability

The dried samples underwent serial dilution, followed by triplicate drop plating on MRS agar. Subsequently, rehydration was carried out to restore the initial solids level using sterile Ringer's solution (No. 15525, Merck, Darmstadt, Germany). Plates were placed in an anaerobic jar (Anaerocults A, Merck, Darmstadt, D) and incubated at 37°C for 48 h (Golowczyc et al. 2010). The survival rate was calculated using the following formula:

(2) $$\%\text{Survival rate}=\left(\frac{N}{N_{0}}\right)\times 100$$

where *N*_0_ represents the number of initially viable cells before drying and *N* was the viable cell count after drying (log CFU/g DM).

#### 2.8.4 Determination of Bulk and Tapped Density

Bulk density was determined by dividing the mass of the powder by the volume it occupied in the cylinder. The cylinder was manually tapped on the surface until there was no further change in volume, allowing the determination of tapped density (Rajam and Anandharamakrishnan 2015).

#### 2.8.5 Determination of Glass Transition Temperature (Tg), FT‐IR Spectroscopy, and Particle Morphology

The Tg of dried probiotic powder was determined using differential scanning calorimetry (DSC, TA DSC Q2000, New Castle, DE, USA). A sample weighing around 6 mg was sealed in an aluminum pan after it was weighed. The samples were heated at a rate of 10°C/min to rise from −40°C to 110°C after being initially chilled from 25°C to −40°C. The analysis obtained the Tg end (Te), Tg (Tg), and initially Tg (T0).

The morphology of the dried probiotic powder was investigated using a scanning electron microscope (SEM, FEI Quanta250 FEG). The samples were mounted separately on aluminum stubs using double scotch tape after being thinly coated with gold using sputter coating.

Using an FTIR spectrophotometer (Perkin Elmer Spectrum Two, Waltham, MA, USA), the interactions between the carrier materials and the impact of various drying techniques were determined, and fingerprint areas were evaluated. At room temperature, the infrared spectra were obtained within the 600–4000 cm^−1^ range, with a spectral resolution of 4 cm^−1^. The data were averaged and shown as the percentage of transmission versus the wavenumber (cm^−1^) after 20 scans.

#### 2.8.6 Statistical Analysis

All results obtained in the effects of different carrier materials for SD section were statistically analyzed by ANOVA and Duncan post hoc test. Statistical package software (SPSS ver. 220, SPSS Inc.; Chicago, IL, USA) was used for statistical analyses. Statistical significance was set at *p* < 0.05.

## 3 Results and Discussion

### 3.1 Effect of Spray‐Drying Process Conditions on Response Variables

**FIGURE 1:**
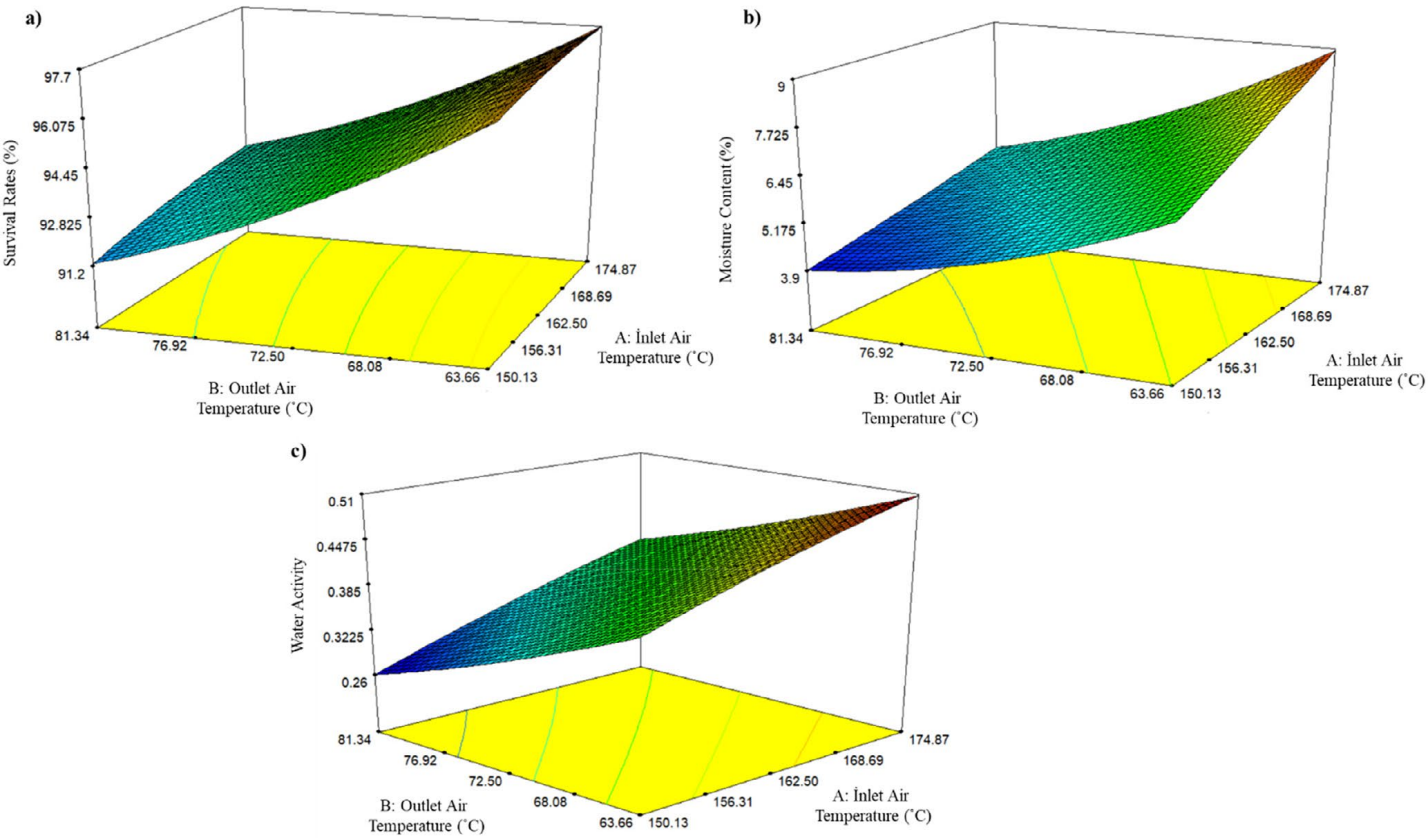
Contour maps indicating the effects of SD independent variables on (a) survival rate (%), (b) MC (%), and (c) water activity of *L. plantarum* BG24 powder.

SD experiments of *L. plantarum BG24* strain were carried out in RSM (20% w/v) and YE (0.5% w/v) as carrier materials at different inlet and outlet air temperatures. Table 1 presents the data on the survival of *L. plantarum* BG24 at various inlet and outlet temperatures during the SD process. Additionally, Table 1 also displays the microbiological viability of *L. plantarum* BG24 before and after SD. The survival rate was observed to range from 92.54% to 99.73% under different process conditions (Table 1). The experimental data showed an excellent fit with a survival rate of *R* ^2^ = 0.9585, indicating a statistically significant second‐order regression model (Table 2). Also, the survival rate model equation is given in Equation (3). It was noted that as the outlet air temperature increased and the inlet air temperature decreased, the survival rate of probiotic bacteria decreased, as depicted in Figure 1a.

(3) $$\text{Survival rate}\;\left(\%\right)=94.29–2.72\times B+0.52\times B^{2}$$

where *B* is the outlet air temperature (°C).

**TABLE 2:** ANOVA results of linear, quadratic, and interaction terms for each response variables for spray‐dried *Lactobacillus plantarum* BG24 powder.

Source	Viable cell counts (log CFU/g DM) before drying		Viable cell counts (log CFU/g DM) after drying		Survival rate (%)		MC (%. wb)		Water activity		Drying yield (%)		Bulk density (kg/m^3^)		Tapped density (kg/m^3^)	
	SS	*p*	SS	*p*	SS	*p*	SS	*p*	SS	*p*	SS	*p*	SS	*p*	SS	*p*
Model	0.13	0.1822	0.65	0.0034*	66.60	0.0001*	32.49	< 0.0001*	0.053	< 0.0001*	138.70	< 0.0001*	29,758.4	0.0053*	18,145.3	0.0681
*A*	5.74 × 10^−4^	0.8364	0.01	0.3490	2.06	0.0606	7.74	< 0.0001*	0.018	< 0.0001*	44.73	< 0.0001*	9600.1	0.0061*	6743.5	0.0389*
*B*	1.27 × 10^−3^	0.7593	0.59	0.0002*	57.31	< 0.0001*	23.75	< 0.0001*	0.034	< 0.0001*	76.65	< 0.0001*	17,428.2	0.0012*	10,293.1	0.0165*
*AB*	9.98 × 10^−2^	0.4013	5.08 × 10^−3^	0.5368	0.10	0.6331	0.27	0.0215*	4.000 × 10^−4^	0.0842	0.87	0.2625	23.5	0.8534	14.6	0.9093
*A* ^2^	2.6 × 10^−2^	0.6621	7.69 × 10^−3^	0.4504	1.88	0.0701	0.10	0.1157	1.830 × 10^−4^	0.2158	6.00	0.0153*	228.5	0.5685	672.2	0.4496
*B* ^2^	0.12	0.0178*	0.04	0.1179	6.01	0.0066*	0.68	0.0023*	7.488 × 10^−6^	0.7911	12.42	0.0025*	2634.5	0.0817	563.8	0.4872
Residual	0.09	—	0.08	—	2.89	—	0.22	—	6.919 × 10^−4^	—	4.12	—	4467.3	—	7339.2	—
Lack of fit	0.03	0.5548	0.03	0.6584	0.80	0.6981	0.14	0.2336	4.436 × 10^−4^	0.2104	3.27	0.0746	2261.5	0.3730	3895.0	0.3412
Pure error	0.05	—	0.06	—	2.09	—	0.083	—	2.484 × 10^−4^	—	0.85	—	2205.8	—	3444.3	—
Total	0.22	—	0.74	—	66.49	—	32.71	—	0.054	—	142.82	—	34,225.8	—	25,484.5	—
*R* ^2^	—	—	—	—	0.9585	—	0.9933	—	0.9871	—	—	—	—	—	—	—
*R* ^2^_adj_	—	—	—	—	0.9288	—	0.9886	—	0.9780	—	—	—	—	—	—	—
C. V. %	—	—	—	—	0.67	—	2.92	—	2.58	—	—	—	—	—	—	—
PRESS	—	—	—	—	8.92	—	1.09	—	3.542 × 10^−3^	—	—	—	—	—	—	—
Adequate precision	—	—	—	—	17.357	—	45.139	—	33.542	—	—	—	—	—	—	—

Abbreviations: *A*, inlet air temperature; *B*, outlet air temperature, significant at 95% level; SS, sum of squares. The asterisk (*) indicates statistically significant results at *p* < 0.05.

This emphasizes the significant influence of the outlet temperature on the ability of probiotic bacteria to survive during the SD process.

As stated in various studies in the literature, outlet temperatures higher than 85°C–90°C are lethal for probiotic microorganisms. This suggests that, as would be predicted, the outlet temperature has a major influence on the cell survival rate (Corcoran et al. 2004; Gardiner et al. 2000; Zamora, Carretero, and Parés 2006). High temperatures not only harm the microorganism's membranes but also impact proteins, DNA, RNA, and ribosomes, ultimately resulting in the loss of the organism's functioning and capacity to survive (Ananta, Volkert, and Knorr 2005; Lapsiri, Bhandari, and Wanchaitanawong 2012). The survival rate (%) increased with increasing inlet air temperature in a study where raspberry juice containing a blend of various probiotics (*L. acidophilus* NRRL B‐4495 and *L. rhamnosus* NRRL B‐442) was dried with spraying (Anekella and Orsat 2013). Even though the droplets in SD are exposed to high temperatures, there is very little chance of them overheating. The spray dryer chamber facilitates rapid drying due to the droplet's expansive surface area and the significant temperature contrast between the droplet and the hot air. This process effectively maintains a low internal temperature for the droplet, thereby preserving the nutritional and sensory qualities of the different food components. The cooling effect is primarily attributed to the evaporation of water (Phisut 2012).

According to our research, we discovered that the viability decreased as the outlet air temperature increased. Out of all the temperatures studied, the lowest outlet temperature of 60°C correlated with the highest survival rate of 99.73% during the SD process. Since the *L. plantarum* BG24 strain exhibited the highest survival rate at all inlet and outlet temperatures examined in this study, it could be evaluated as highly resistant to drying and high temperatures. However, our results also highlighted the importance of optimizing drying conditions to ensure safe storage. In addition to a high survival rate, it is crucial to maintain low MC and *a*_w_ for safe storage.

An important quality aspect of probiotic powder products is their MC. Probiotic cell viability during storage, powder flow characteristics, and *a*_w_ are all influenced by the product MC. Low MC powders provide greater stability and have extended shelf life (Guerin et al. 2017). In this study, the MC and *a*_w_ values of SD probiotic (*L. plantarum* BG24) powders were found to range from 3.79% to 9.3% (wb) and 0.29–0.51, respectively (Table 1). It is recommended that the MC and water activity value of powder products should be kept below 5% and 0.25%, respectively, if long‐term storage is the target (Teixeira et al. 1995). It was determined that the MC of spray‐dried probiotic powders decreased with a decrease in the inlet air temperature and an increase in the outlet air temperature (Figure 1b). The MC model equation is given in Equation (4).

(4) $$\mathrm{MC}\;\left(\%\right)=5.68+0.95\times A-1.55\times B-0.26\times \mathrm{AB}+0.43\times B^{2}$$

where *A* is the inlet air temperature (°C) and *B* is the outlet air temperature (°C).

When the inlet air temperature increases, hot drying air at inlet initially contacts the probiotic solution. This can cause the surface to dry quickly, forming a crust on the surface of the droplets. The crust can trap the moisture inside, preventing it from escaping, which can lead to higher MC and water activity in the final powder product. At lower inlet temperatures, crust formation is less pronounced. Higher outlet temperatures, on the other hand, allow the moisture within the product to evaporate more effectively and be expelled, resulting in lower MC and water activity in the final product (Gharsallaoui et al. 2007; Masum et al. 2020). Additionally, adjusting the feed flow rate (either increasing or decreasing) during SD is possible to reach the target outlet temperature. This adjustment can either increase or decrease the MC (Kim, Chen, and Pearce 2009). According to Khalaf, Mahmmad, and Al‐muhsin (2006), the MC of milk powders increased with rising inlet air temperature. The outlet air temperature regulates the final MC of the powder; the higher the outlet air temperature, the lower the final MC and vice versa (Gaiani et al. 2011; Khalaf, Mahmmad, and Al‐muhsin 2006). In a similar study, Masum et al. (2020) reported that the MC increased with rising inlet air temperature.

The *a*_w_ is a crucial quantity for SD powders since it affects the product's shelf life. In this study, the *a*_w_ of SD probiotics decreased with increasing outlet temperature and decreasing inlet temperature (Figure 1c), according to findings previously reported for a number of probiotic powders (Desmond, Stanton, et al. 2002; Teixeira et al. 1995). The water activity model equation is given in Equation (5).

(5) $$a_{w}=0.39+0.056\times A-0.068\times B$$

where *A* is the inlet air temperature (°C) and *B* is the outlet air temperature (°C).

In a similar manner, another study found that water activity increased with rising inlet air temperature, while it decreased with higher outlet air temperature (Masum et al. 2020). According to Vardin and Yasar (2012), rising *a*_w_ means that there is more free water accessible for deteriorative processes, which reduces shelf life. When the *a*_w_ is < 0.2, several studies indicated that dried microorganisms can be stored for an extended period of time (Teixeira et al. 1995). While there may be a relationship between a food's *a*_w_ and its overall MC, this relationship is not always present (Bicudo et al. 2015). In this study, the lowest *a*_w_ value obtained was 0.29. Therefore, survival of this strain could be improved under controlled *a*_w_.

### 3.2 Effect of Spray‐Drying Process Conditions on Drying Yield and Powder Density

Drying conditions significantly affect the drying yield throughout the SD, with factors such as inlet and outlet temperatures, feed rate, airflow rate, and humidity playing crucial roles. Higher inlet temperatures can increase drying efficiency by rapidly removing moisture, leading to higher yields. However, excessively high temperatures can cause thermal degradation of heat‐sensitive components, reducing the overall yield and quality. The outlet temperature must also be optimized to ensure sufficient moisture removal without over‐drying of the product. The feed rate affects the amount of material entering the dryer; a higher feed rate can increase yield but may result in incomplete drying if the residence time is insufficient. The air flow rate influences the drying kinetics by determining the rate at which moisture‐laden air is removed from the drying chamber. Higher air flow rates can enhance moisture removal, increasing yield, but may also lead to powder loss through the exhaust system. Humidity levels impact the drying efficiency, with lower humidity promoting faster moisture removal and higher yields. However, extremely low humidity can cause excessive drying and particle aggregation. Understanding the interplay of these factors and optimizing them is essential for maximizing SD yield while maintaining product quality (Tontul and Topuz 2017).

An essential factor for SD powders is the drying yield. The drying yield in the present study ranged from 68.94% to 80.52% based on the inlet and outlet temperatures (Table 1). The highest powder yield (80.52%) was obtained at 150°C of inlet air temperature and 81°C of outlet air temperature (Table 1). It was stated that the drying yield % decreased with increasing inlet temperature and increased with increasing outlet temperature (Table 2). Since a higher yield corresponds to more benefits, it is a crucial indication for the powder industry. The food components' stickiness issue is the primary cause of the low product yield (Can Karaca, Guzel, and Ak 2016). In order to achieve an effective SD, Bhandari, Datta, and Howes (1997) stated that the product yield must be more than 50%.

The average bulk densities of powdered food products with desired flow properties range from 200 to 800 kg/m^3^, while tapped density values range from 300 to 2000 kg/m^3^ (Himmetagaoglu and Erbay 2019). In this study, the bulk density values of samples dried at different inlet and outlet air temperatures vary between 265.39 and 416.77 kg/m^3^, while the tapped density values range from 377.93 to 507.44 kg/m^3^ (Table 1). The bulk and tapped density values obtained within these ranges indicate that the flow properties of the produced powders are favorable. The bulk density and tapped density values decreased with increasing inlet temperature and increased with increasing outlet temperature (Table 2). The lowest bulk density (235.53 kg/m^3^) and tapped density (320.03 kg/m^3^) values were found in the sample dried at 175°C of inlet temperature and 64°C of outlet temperature. Similarly, in a study in which *L. acidophilus* (NCDC 016) culture was dried by SD, it was reported that bulk density decreased with increasing air temperature (Arepally and Goswami 2019). Low bulk density and porous powders exhibit better reconstitution behaviors compared to high bulk density powders, while powders with higher bulk density and lower porosity have been reported to have increased storage stability (Premi and Sharma 2017). According to Goula and Adamopoulos (2010), there is a correlation between increasing inlet temperature and a decrease in bulk density because of faster evaporation rates that lead to a more porous structure. Walton (2000) stated that as the temperature of the drying air increases, bulk and particle densities usually decrease and the particles become more amorphous.

### 3.3 Optimization of Spray‐Drying Process Conditions

Using the desirability function technique and superimposing approaches aimed at maximizing survival rate and minimizing *a*_w_ and MC, the inlet air temperature (*A*; 145°C–180°C) and outlet air temperature (*B*; 60°C–85°C), chosen as the independent variables for SD of *L. plantarum* BG24, were optimized. The optimum point was found by using the second‐order polynomial models that were derived for each response. Using the desirability function technique, the optimum temperature was determined to be 150°C for the inlet and 83°C for the outlet, with a desirability point of 1.0. Under these optimized process conditions, MC, *a*_w_, and survival rate were estimated as 3.57%, 0.266%, and 92.53%, respectively. The optimization study excluded other characteristics of the samples generated using the CCRD experimental methodology.

Experimental verification of the predicted optimum point was carried out, and Table 3 shows the average results of the five validation experiments agreed with the predictions. To determine whether there was a statistically significant difference between the average results obtained from the optimum point validation trials and the values predicted by the model, a one‐sample *t*‐test was carried out for each response. As a result, the survival rate was found to be greater than the expected value (*p* < 0.05), while the difference between the predicted and experimental values in terms of *a*_w_ and MC was not statistically significant (*p* > 0.05).

**TABLE 3:** Results of statistical analysis for verification of the optimization results.

Response	Predicted value	Experimental value^a^	SE	Differences	% Error^b^	*p*
Survival rate (%)	92.23	93.16 ± 0.50	0.225	0.93	1.00	0.014
MC (%, wb	3.57	3.60 ± 0.08	0.038	0.04	0.72	0.394
Water activity	0.266	0.269 ± 0.004	0.002	0.003	0.97	0.186

Abbreviation: SE, mean standard error.

^a^ Experimental values were given as mean ± SD.

^b^ % Error = (|*y* exp − *y* pre|/*y* exp) × 100.

### 3.4 The Effect of Different Carrier Materials During Spray Drying

After optimizing the SD process conditions, SD experiments of *L. plantarum* BG24 were carried out at the optimum point (150°C inlet and 83°C outlet temperatures) using different carrier materials in order to obtain the highest survival rate and the lowest MC and *a*_w_ for dried probiotic bacteria. For this purpose, *L. plantarum* was suspended in distilled water dispersions with 20% (w/v) of each of the following carrier materials.

A compatible and encapsulating matrix (RSM; RSM + GA; RSM + MD (DE18–20); RSM + MD (DE 4–7); RSM + SUC; SPI; SPI + MD (DE18–20); SPI + MD (DE 4–7); SPI + LAC; SPI + SUC); and a hydrophilic water soluble compound capable of replacing, at least partially, the removed water or hydrogen bonding and of forming a glassy barrier (MD, SUC, LAC) were created by combining a protein source (RSM and SPI) with a carbohydrate (the protein: carbohydrate ratio was 1:1).

Viable counts of LAB strain, *a*_w_, MC, survival rate, drying yield, and T_g_ were analyzed on samples dried using different carrier materials, and the results are given in Table 4.

**TABLE 4:** Viable cell counts, survival rate, water activity, MC, drying yield, and glass transition temperature, Tg of *Lactobacillus plantarum* BG24 after being spray dried with different carrier materials at optimum process conditions.

Carrier materials for drying	Viable cell counts (log CFU/g DM)	Viable cell counts (log CFU/g DM)	Survival rate (%)	MC (%, wb)	Water activity (aw)	Drying yield (%)	Glass transition temperature (Tg°C)
	Before drying	After drying					
RSM	9.48^f^ ± 0.13	8.83^g^ ± 0.14	93.16	3.60^a^ ± 0.08	0.269^ab^ ± 0.004	80.52	43.81^d^ ± 0.67
RSM + MD (DE: 18–20)	9.34^bc^ ± 0.03	6.94^a^ ± 0.04	74.32	3.89^bc^ ± 0.14	0.258^a^ ± 0.002	80.46	50.67^i^ ± 0.16
RSM + MD (DE: 4–7)	9.44^c^ ± 0.14	7.80^c^ ± 0.02	82.62	4.17^de^ ± 0.06	0.258^a^ ± 0.003	79.83	50.53^ı^ ± 0.43
RSM + GA	9.66^ef^ ± 0.01	8.85^g^ ± 0.02	91.63	5.15^f^ ± 0.19	0.311^d^ ± 0.003	51.59	47.13^g^ ± 0.21
RSM + SUC	9.58^de^ ± 0.01	8.66^f^ ± 0.10	90.47	5.01^f^ ± 0.16	0.326^e^ ± 0.001	79.22	33.95^a^ ± 0.34
SPI	9.86^g^ ± 0.06	9.13^h^ ± 0.04	92.58	6.28^g^ ± 0.14	0.346^f^ ± 0.003	52.46	43.36^c^ ± 0.03
SPI + MD (DE: 18–20)	9.50^d^ ± 0.05	6.90^a^ ± 0.11	72.60	4.47^e^ ± 0.25	0.279^b^ ± 0.004	74.27	47.08^f^ ± 0.02
SPI + MD (DE: 4–7)	8.92^a^ ± 0.10	7.46^b^ ± 0.04	83.60	5.89^g^ ± 0.30	0.303^cd^ ± 0.005	35.53	45.17^e^ ± 0.22
SPI + LAC	9.45^cd^ ± 0.08	8.36^e^ ± 0.03	88.45	4.03^cd^ ± 0.09	0.298^c^ ± 0.004	68.79	49.53^g^ ± 0.36
SPI + SUC	9.76^f^ ± 0.04	8.15^d^ ± 0.05	83.54	3.64^ab^ ± 0.24	0.29^3c^ ± 0.004	58.42	41.17^b^ ± 0.73

*Note:* Data were expressed as mean value ± SD (*n* = 2). Mean values with different letters in line are significantly different (*p* < 0.05).

Abbreviations: DE, dextrose equivalent; GA, gum Arabic; LAC, lactose; MD, maltodextrin; RSM, reconstituted skim powder; SPI, soy protein isolate; SUC, sucrose.

The survival of *L. plantarum* BG24 and MC and *a*_w_ values were lower in the powders when RSM with MD was used as carriers. The survival rate (93%) was higher when RSM alone was used as a carrier, while the reduction log cycle was significantly lower (*p* < 0.05) when RSM was supplemented with MD (Table 4). Our results confirmed those of Desmond, Ross, et al. (2002), who observed RSM alone or RSM + GA for *L. plantarum* to improve viability. MD exhibited poor results, whereas GA and SUC with RSM offered very good protection. SPI was acceptable when used alone but not effective in combination with MD, LAC, and SUC.

In a study, whey permeates (which was mostly lactose) and RSM were used as drying agents for drying *L. acidophilus* using a spray dryer, and the viability of cells dried using whey permeate was found to be half that of cells dried using RSM. This result was explained since the proteins in RSM stabilized the components of the cell membrane, thereby preventing damage to cells. Additionally, it was concluded that they can create a protective layer on the bacterial cell wall through their interaction with milk calcium (Huang et al. 2014; Zheng et al. 2016). In another study, which used an RSM as a drying agent to dry *Lacticaseibacillus paracasei*, *L. paracasei* A13, and *L. acidophilus* A9 cultures, the survival rate of strains after drying was found to be approximately 100% (Páez et al. 2013).

On the other hand, *a*_w_ values of samples dried in RSM were found to be higher than those of samples dried in SPI or combinations (*p* < 0.05). The maximum drying yield was obtained for probiotic powders dried in RSM or RSM combinations, whereas SPI gave a lower drying yield.

Tg is a thermodynamic characteristic of materials that changes when water is present. A glassy state matrix is more resistant to harmful processes because mass transfer rates are much slower there. It is found that Tg is higher in samples where RSM is used as the carrier material, meaning that the environment's structure is maintained until the higher temperature limit without changing into a viscous structure (Table 4). The use of SPI as the drying agent results in a decrease in Tg, as shown in Table 4, when SPI + MD and RSM + MD are compared. Furthermore, it is interesting that Tg is greatly lowered when SUC is used as the drying agent.

**FIGURE 2:**
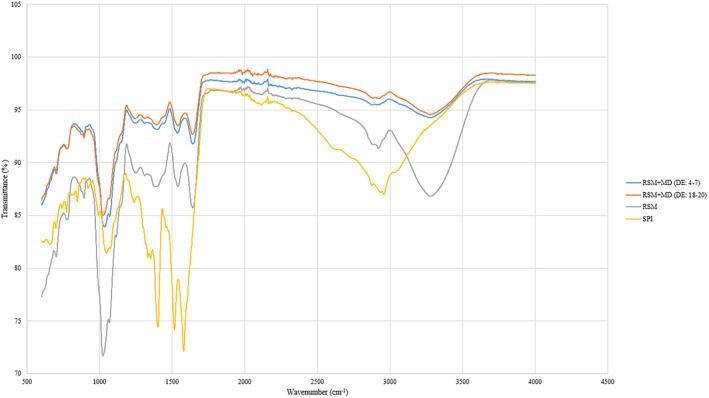
FTIR spectra of spray‐dried probiotic powders with different carrier materials (RSM, RSM + MD, and SPI). MD, maltodextrin; RSM, reconstituted skim milk; SPI: soy protein isolate.

The effects of several carrier materials on the SD of the *L. plantarum* BG24 strain were investigated using FTIR spectroscopic analysis. Figure 2 shows the FTIR spectra of dried probiotic powders that were produced under optimum SD process conditions using RSM, RSM + MD (DE 18–20), RSM + MD (DE 4–7), and SPI as drying agents. FTIR analysis was not performed since the probiotic powders dried with other carrier materials used in this study exhibited lower survival rates, greater MC, and *a*_w_ values. FTIR analysis was carried out on probiotic powders that had been dried using SPI, despite the fact that it is well known that the processing outputs obtained this way are worse than those obtained using RSM. The results showed that the FTIR spectrum is affected by the carrier materials utilized during drying.

The FTIR spectrum in the drying process with RSM, RSM + MD, and SPI carrier materials was found to be similar to the spectrums of the carrier materials themselves. This result demonstrates the effectiveness of the carrier materials utilized. The magnitude of the peak at 3600 cm^−1^ wavelength, which represents the O‐H bond, also expresses the MC, which is significant for powder goods (Azizi et al. 2021; Du et al. 2019). Samples containing only RSM and SPI as drying agents have greater MCs than RSM + MD, as shown in Figure 2.

Additionally, the peaks observed in the 1000–1500 cm^−1^ region are primarily associated with carbohydrate structures, including C‐O and C‐H bending vibrations (Du et al. 2019). RSM + MD (DE 18–20) and RSM + MD (DE 4–7) show more pronounced peaks in this region compared to RSM and SPI, indicating a higher carbohydrate content in MD‐containing samples. This corresponds to the expected higher carbohydrate content due to the presence of MD.

Amide I Region (1600–1700 cm^−1^) is characteristic of protein secondary structures, mainly the C=O stretching vibrations of the peptide bonds in proteins (Ye et al. 2017). All samples show peaks in this region, indicating the presence of proteins. However, the intensity of these peaks varies, with RSM and SPI showing more prominent peaks compared to RSM + MD mixtures. This suggests that the pure RSM and SPI samples have a higher protein content or more pronounced protein structures. Also, Amide II Region (1500–1600 cm^−1^) is associated with N‐H bending and C‐N stretching vibrations. Similar to the Amide I region, peaks in the Amide II region are more prominent in RSM and SPI samples, reinforcing the higher protein content in these carriers compared to the RSM + MD mixtures. Peaks in this region are indicative of C‐H stretching vibrations (2800–3000 cm^−1^) from CH_2_ and CH_3_ groups, common in both lipids and carbohydrates (Botros et al. 2013). The spectra for all samples show peaks in this region, but RSM + MD samples tend to have slightly higher intensities, reflecting the higher carbohydrate and potential lipid content in MD‐containing samples.

In summary, the FTIR spectra demonstrate the distinct influence of different carrier materials on the chemical composition of dried probiotic powders. The variation in peak intensities and positions across different wavelength regions underscores the differences in protein, carbohydrate, and MC introduced by each carrier material. This detailed spectral analysis confirms the suitability of these materials for maintaining the integrity and functionality of the dried probiotics.

### 3.5 Freeze Drying of *L. plantarum* BG24 and Comparison With SD

After selecting the best protective agent giving the highest survival rate (RSM with YE) for the probiotic suspension during the SD at optimum process conditions of 150°C inlet and 83°C outlet temperature, the suspension prepared with the same formulation was freeze dried at 0.15 mbar for 18 h. Table 5 shows the *a*_w_, MC, and survival rate values besides Tg of freeze‐dried and SD samples. Because probiotic cells are subjected to high temperatures and quick drying during the SD process, it is clear that the approach yielded poorer cell survival than the FD method. This is because thermal injury may be the primary source of cell damage in probiotic bacteria (Broeckx et al. 2016).

**TABLE 5:** Influence of drying methods on water activity, MC, viable cell counts, survival rate, and glass transition temperatures of *Lactobacillus plantarum* BG24 powders.

	SD (150°C/83°C)	FD (0.15 mbar, 18 h)
Viable cell counts (log CFU/g DM)	8.83 ± 0.14	9.21 ± 0.08
Survival rate (%)	93.16 ± 0.50	97.69 ± 0.14
MC (%, wb)	3.60 ± 0.08	5.10 ± 0.19
Water activity (aw)	0.269 ± 0.004	0.189 ± 0.03
Glass transition temperature (°C)	43.81 ± 0.67	50.29 ± 0.08

Table 5 also shows that *a*_w_ values of SD powders are higher than freeze‐dried ones (*p* < 0.05) on the contrary of MCs. This can be attributed that the longer contact with air and the higher hygroscopicity of the freeze‐dried product caused the higher MC. The loss of bound water at the cell surface after SD is attributed to damage to probiotic bacteria in addition to the heat impact (Santivarangkna, Higl, and Foerst 2008). Water is crucial for the integrity and stability of probiotic cells; its absence can seriously harm the cell wall, membrane, and surface proteins, reducing the cells' ability to survive after drying (Castro, Teixeira, and Kirby 1997; Teixeira, Castro, and Kirby 1995). Furthermore, FD is a well‐known and popular technique for drying of probiotics, although it has a number of drawbacks. The technique is expensive and time‐consuming, and the finished product is a dry cake. In order to obtain individual powder particles, therefore, one more processing step is required (Maltesen and van de Weert 2008; Santivarangkna, Higl, and Foerst 2008). The higher Tg of dried microorganisms provides better preservation of viability, especially during storage (Conrad et al. 2000). It is seen that, in parallel with the use of hot air in the SD process, the Tg of the SD powders is lower than that of freeze‐dried ones (Table 5).

**FIGURE 3:**
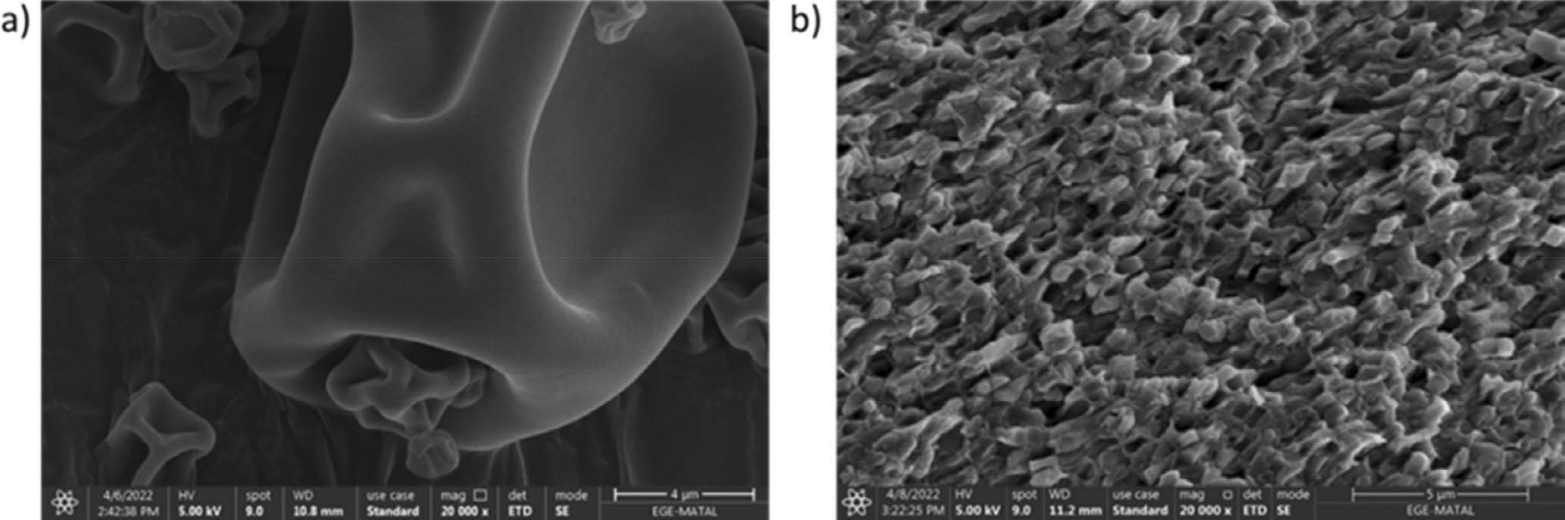
SEM (×20,000) images of probiotic powders obtained (a) spray dried at optimum conditions and (b) freeze dried.

Figure 3 shows 20,000 times magnified SEM images of the powders obtained by SD and FD methods.

Powders produced using the FD technique are amorphous and have a porous structure resembling flakes. On the other hand, the surface of SD powders is concave, indented, and wrinkled, giving them a hemispherical form (Sharifi et al. 2021). Haque and Roos (2006) encapsulated a probiotic strain using a combination of whey protein + lactose using SD and found that the surfaces of the powders were hemispherical, smooth, and concave. This result was interpreted as the use of high temperatures in the moisture loss phase, and the rapid evaporation of moisture caused the formation of voids on the surface of the powders.

### 3.6 Storage Stability of Probiotic Powders

**FIGURE 4:**
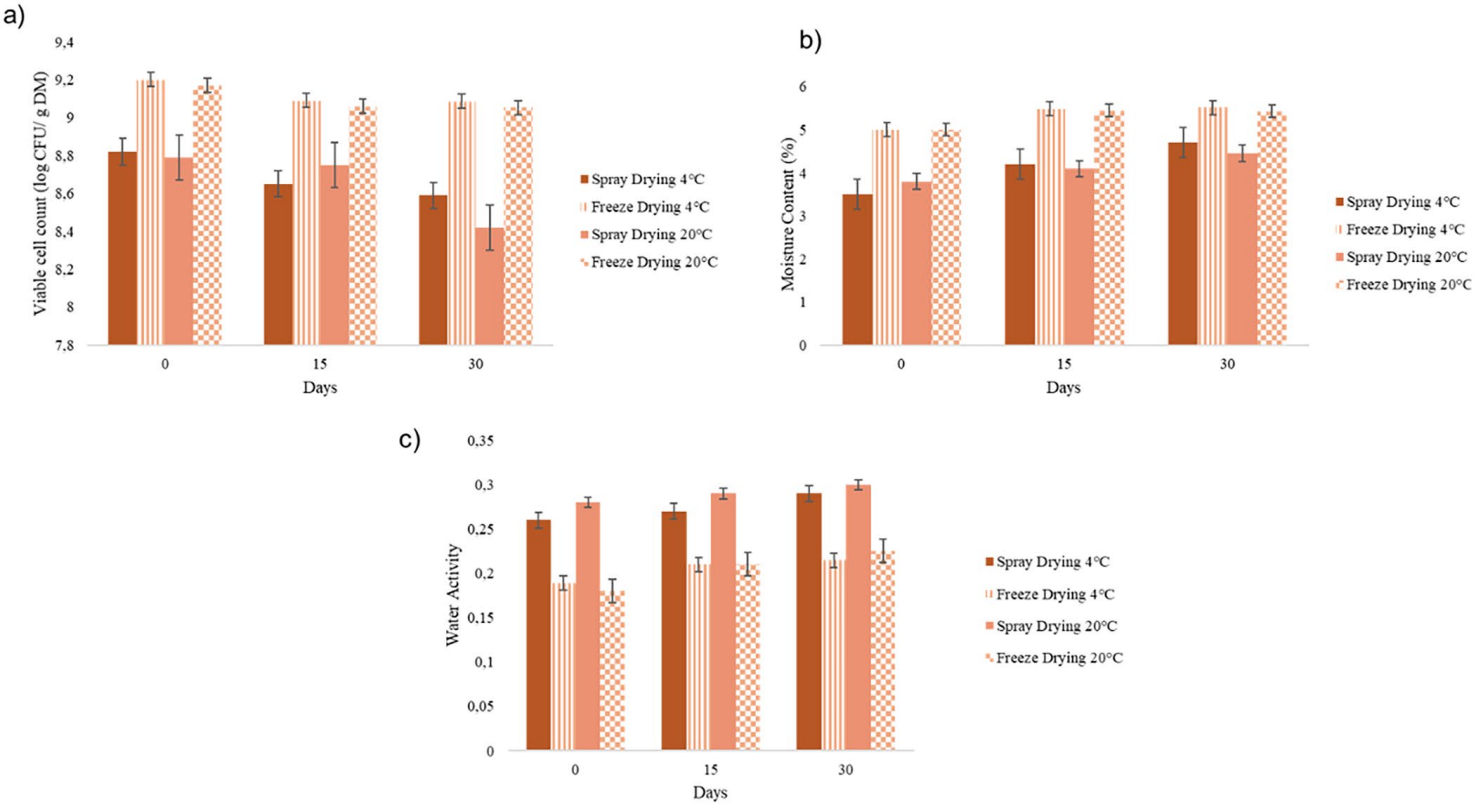
(a) Viable cell count, (b) MC, (c) water activity of spray‐dried and freeze‐dried *Lactobacillus plantarum* BG24 during storage at different temperatures; refrigerator condition (4°C) and room temperature (20°C).

Storage stability of probiotic powders obtained by SD and FD was evaluated under two different storage temperatures (Refrigerator (+4°C) and room temperature (20°C)) in terms of viable cell count, *a*_w_ and MC. Figure 4a shows the survival of *L. plantarum* BG24 powders during storage up to 30 days at 4°C and 20°C.

While there was a 1% decrease in cell counts of the samples dried with FD during storage, it was determined that there was a 3%–5% decrease in cell counts of the samples dried by spraying. As seen in Figure 4a, freeze‐dried samples showed a higher survival rate after 30 days of storage.

Furthermore, it is interesting to note that the FD probiotic powder has a higher MC (Figure 4b). From this, it can be inferred that the bacteria were more likely to survive in an environment with higher MC. SD powder, on the other hand, produces a lower MC and shows poor cell survival during storage. These findings confirm that a previous study (de Valdez et al. 1985) found that LAB survival decreased with increasing dehydration and that optimal MC is necessary to preserve cell survival during storage (Zayed and Roos 2004).

According to previous research studies (Desmond, Ross, et al. 2002; Gardiner et al. 2000; Teixeira et al. 1995), our findings indicated that the microorganisms' survival rate was higher at lower storage temperatures for both dried samples, indicating that temperature was a crucial factor.

The viability of FD probiotics remained relatively stable during the 30 days of storage period at 4°C. However, at 20°C, there was a more noticeable decrease in viability, particularly for SD samples. It is seen that the survival loss is greater at 20°C for SD powders. The relationship between storage temperature and probiotic viability in powder form is inversely proportional (Barbosa et al. 2015; Corcoran et al. 2004). Initially, there were a significant number of viable microorganisms, specifically *Lactobacillus*, in the SD powder. However, as the probiotic bacteria in the powders were stored, these numbers gradually decreased. From an economic standpoint, a survival rate of < 10% would be deemed unacceptable. On the contrary, the MC and *a*_w_ (Figure 4c) values of the probiotic powders slightly increased during storage.

Studies by Silva et al. (2002) and Wang, Yu, and Chou (2004) found that probiotic powders stored at room temperature were less stable than samples stored in cold climates. Tang et al. (2020) used SD and FD processes to dry the *L. acidophilus* FTDC 3081 strain, and the dried powders were kept in the refrigerator (4°C) and at room temperature (20°C). According to the findings of this study, the survival rate of FD samples was higher than that of SD samples at both initial and during storage. When the storage conditions were compared, the survival rate stored in the refrigerator (4°C) in both drying techniques was greater during storage.

## 4 Conclusion

SD was employed to manufacture *L. plantarum* BG24 powder at different inlet and outlet air temperatures. The process parameters were optimized using the response surface method to achieve maximum cell viability while minimizing MC and *a*_w_. The optimal conditions were found to be an inlet temperature of 150°C, an outlet temperature of 83°C, a survival rate of 92.23%, a MC of 3.57%, and an *a*_w_ of 0.266. When the appropriate carrier materials were used, *L. plantarum* BG24 demonstrated resilience during SD. RSM (20% w/v) and YE (0.5% w/v) resulted in a high number of viable cells after SD, meeting the requirement of 8–9 log CFU/g DM of powder. The use of RSM + GA and SPI as drying agents provided effective protection for the probiotic bacteria. A comparison between SD and FD revealed that FD had a higher viability rate (97.69%) but also a higher MC. Furthermore, the study demonstrated that reducing the moisture level had a negative impact on cell viability. In addition to the drying technique used, the temperature at which the *L. plantarum* BG24 powder was stored also affected its viability. Storing the probiotic powder at lower temperatures resulted in higher cell viability for both FD and SD powders.

This study demonstrated that optimizing SD conditions can produce probiotic powders with high cell viability and low MC. Future studies could focus on the investigation of new drying techniques, such as ultrasonic drying, microencapsulation, and combined drying methods, to assess their impact on probiotic stability and viability. The response of different probiotic strains to SD and FD processes should be examined to identify which strains perform better under specific conditions. Furthermore, exploring the industrial applications of probiotic powders, including their use in food, pharmaceutical, and healthy products, and evaluating their long‐term effects on consumer health are essential.

Its potential for enhancing functional foods and beverages is significant, as it could be integrated into a range of probiotic products, including dairy items such as yogurt and kefir, as well as plant‐based alternatives like soy or almond‐based yogurts and fermented drinks. Probiotic properties of *L. plantarum* BG24 could contribute to improved gut health and immune function, making it an attractive addition to health‐oriented food products. Furthermore, *L. plantarum* BG24 holds potential in the fermentation of vegetables, fruits, and grains, potentially improving their nutritional value, taste, and preservation. Beyond food, this strain may find applications in the development of new dietary supplements and therapeutic products due to its beneficial microbiological properties. Continued research and development could reveal additional uses in biotechnology and agriculture, highlighting the versatile applications of this novel strain.

Finally, understanding consumer acceptance, market potential, and marketing strategies for probiotic powder products will be important for expanding their commercial applications. These suggestions could drive innovation in probiotic powder production, enhance the knowledge base in this field, and contribute to the broader commercialization of probiotic products. Moreover, expanding this research to include a broader range of probiotic strains could pave the way for the development of diverse probiotic products tailored to specific health needs. Overall, the findings of this study contribute valuable insights into the SD process for probiotics, offering a foundation for future innovations and applications in the field of probiotic manufacturing.

## Author Contributions

**Figen Kaymak Ertekin:** conceptualization (equal), project administration (equal), supervision (equal), writing – review and editing (equal). **Özgün Köprüalan Aydın:** conceptualization (equal), formal analysis (equal), writing – original draft (equal), writing – review and editing (equal). **Özgül Altay:** conceptualization (equal), formal analysis (equal), writing – original draft (equal), writing – review and editing (equal).

## Conflicts of Interest

The authors declare no conflicts of interest.

## Data Availability

All data generated or analyzed during this study are included in this published article.
